# Depressive Symptoms and Loneliness Among Black and White Older Adults: The Moderating Effects of Race

**DOI:** 10.1093/geroni/igaa057.077

**Published:** 2020-12-16

**Authors:** Harry Taylor, Ann Nguyen

**Affiliations:** 1 Duke University Medical Center, Durham, North Carolina, United States; 2 Case Western Reserve University, Cleveland, Ohio, United States

## Abstract

Loneliness is consistently linked to worse depression/depressive symptoms; however, few studies examined if this relationship varies by race. The purpose of this study was to determine if race moderated the relationship between loneliness and depressive symptoms among a nationally representative sample of older Black and White adults. Data come from the 2014 wave of the Health and Retirement Study (HRS) Core survey and Psychosocial Leave Behind Questionnaire; only Black and White older adults were included in the analysis (N=6,469). Depressive symptoms were operationalized by the CESD; however, the ‘felt lonely’ item was removed given concerns with collinearity. Loneliness was operationalized using the Hughes 3-Item Loneliness Scale. Sociodemographic variables included gender, age, education, household income, employment status, marital status, and living alone or with others. Furthermore, social support and negative interactions from family members and friends, and religious service attendance were included in the analysis. Lastly, we created an interaction term with race and loneliness. All analyses used survey weights to account for the complex multistage sampling design of the HRS. Missing data were multiply imputed. Older Blacks had higher rates of loneliness and depressive symptoms compared to older Whites. In multivariate analysis, we found race significantly moderated the relationship between loneliness and depressive symptoms while controlling for sociodemographic, social support, negative interaction, and religious attendance covariates. For both older Blacks and Whites, greater loneliness affected depressive symptoms; however, the effect was stronger among Whites than it was for Blacks. Findings can be used to create racially sensitive depression interventions.

